# Male and female residents in postgraduate medical education – A gender comparative analysis of differences in career perspectives and their conditions in Germany

**DOI:** 10.3205/zma001130

**Published:** 2017-11-15

**Authors:** Stine Ziegler, Thomas Zimmermann, Lea Krause-Solberg, Martin Scherer, Hendrik van den Bussche

**Affiliations:** 1 Universitätsklinikum Hamburg-Eppendorf, Institut für Allgemeinmedizin, Hamburg, Germany

**Keywords:** Postgraduate medical education, gender roles, hospital, working time, self-efficacy

## Abstract

**Aim: **This article focuses on the gender-specific career differences of residents in their postgraduate medical education in Germany. In particular the structural obstacles female physicians have to overcome during residency are investigated. Moreover, the study examines the position preferences of male and female physicians in the hospital and in how far occupational self-efficacy corresponds to the interest in a hospital leading position.

**Methods: **The KarMed-Study’s database consists of annual postal surveys throughout the entire residency of medical students, who were in their “Practical Year” in 2008/2009. Descriptive statistics and regression models were used in the analysis.

**Results: **Male and female physicians differ in terms of their preferred work place (hospital, ambulatory care, others), hospital position and working hours. Female physicians prefer part-time work and rarely assume leading positions compared to male physicians. In addition, female physicians, especially those with children, need more time to complete their postgraduate training. Female physicians with children are burdened and disadvantaged more often than their female colleagues without children as well as male physicians in general (e.g. belated start and completion of residency, lower rate of doctorate titles, higher quota of part-time contracts, short-term employment contracts, and higher rates of residency interruption or termination). Besides gender and doctorate title, the occupational self-efficacy expectation has an influence on the preference of leading positions in hospitals. Respondents with a low occupational self-efficacy score are less likely to strive for leading positions with more responsibilities than those with a high score.

**Conclusion: **The results demonstrate clear gender disparities in postgraduate training. Female physicians, especially those with children, are disadvantaged in various areas when compared with their male colleagues. In particular, the low rate of doctorate titles and the low score of self-efficacy expectation are associated negatively with the willingness to aim at leading positions in hospitals. Special measures and programmes should be developed for female physicians to counteract these differences.

## Zusammenfassung

**Zielsetzung: **Der Artikel befasst sich mit den geschlechtsspezifischen Karriereunterschieden von Ärztinnen und Ärzten in der fachärztlichen Weiterbildung. Untersucht wird, welche strukturellen Hindernisse insbesondere die Ärztinnen in der Weiterbildung erleben. Zudem soll nach Unterschieden zwischen den Geschlechtern bezüglich der Positionspräferenz im Krankenhaus gesucht werden. Ferner wird geprüft, inwiefern die berufliche Selbstwirksamkeit mit dem Interesse an einer leitenden Position im Krankenhaus nach der Weiterbildung korrespondiert.

**Methodik: **Datenbasis der Studie sind die jährlichen postalischen Befragungen einer Kohorte von PJ-Studierenden des Jahrgangs 2008/09, die während der gesamten Weiterbildungsdauer verfolgt wird. Für die Auswertung wurden deskriptive Statistiken und Regressionen angewandt.

**Ergebnisse: **Ärztinnen und Ärzte unterscheiden sich hinsichtlich ihrer Präferenzen für Versorgungssektor, Position und Arbeitszeit nach der fachärztlichen Weiterbildung: Ärztinnen präferieren weit öfter als Ärzte eine Arbeit in Teilzeit und möchten weniger oft Leitungspositionen im Krankenhaus übernehmen. Zudem brauchen Ärztinnen meist länger für die Weiterbildung, insbesondere dann, wenn sie Mutter sind. Ärztinnen mit Kind sind auch in anderen Bereichen stärker belastet und benachteiligt als ihre Kolleginnen ohne Kind sowie männlichen Kollegen generell (verspäteter Weiterbildungsbeginn und -abschluss; geringere Promotionsrate; höhere Quote an Teilzeitverträgen, kürzere Arbeitsverträge, Unterbrechungs- und Abbruchraten). Neben Geschlecht und Promotion hat die berufliche Selbstwirksamkeitserwartung (BSWE) einen Einfluss auf die Präferenz von Leitungspositionen im Krankenhaus – Befragte mit einem geringen BSWE-Score streben seltener Leitungspositionen an als jene mit hoher BSWE.

**Schlussfolgerung:** Die Ergebnisse zeigen klare geschlechterspezifische Unterschiede in der fachärztlichen Weiterbildung. Ärztinnen sind in verschiedenen Bereichen stärker belastet als Ärzte, insbesondere Ärztinnen mit Kind. Speziell die geringere Promotionsrate und der BSWE-Score der Ärztinnen sind negativ mit der Bereitschaft assoziiert, eine Leitungsposition im Krankenhaus zu übernehmen. Um diesen Differenzen zu begegnen, sollten spezifische Maßnahmen und Programme für Ärztinnen entwickelt werden.

## 1. Introduction

Over the last four decades, gender lost its impact as a crucial cultural and social identification, egalitarian values have spread and an increasing number of women are working. Nevertheless, the vertical segregation within organisations remains. Leading positions are more often held by men whereas women rather work on lower hierarchical levels [1]. This also applies to the medical sector: in recent years, the percentage of female physicians has increased significantly in almost all OECD countries. In Germany, approximately two thirds of medical graduates are women [2]. However, women still face greater challenges regarding their professional careers. The increase in female physicians is still not represented in all hierarchical positions: only 10% of leadership positions in the medical field in Germany are currently held by women [3]. The high proportion of female physicians causes new challenges in medical care, especially in hospitals [4]. There is an increasing gap between the demand for medical professionals and those looking for work. This intensifies the ongoing discussion about the impact of trends like ‘feminisation’ and ‘Generation Y’ on the medical labour market.

Several studies report differences between male and female physicians regarding their professional plans for the future. Aspects such as regular working hours, predictability and room for private life are more important for women when choosing a specialty and a work environment than for their male counterparts. Alas, women still have major problems reconciling work and family life [5], [6], [7], [8], [9], [10], [11], [12], [13].

The KarMed-Study, run by the Institute of Primary Medical Care of the University Medical Center Hamburg-Eppendorf, surveyed male and female residents about their plans for their professional future over a period of seven years. The project’s main goals include:

- Describing the realistic course of medical careers between achieving a medical license (‘Approbation’) and being recognised as a medical specialist (‘Facharztanerkennung’)
- Summing up factors promoting and inhibiting the achievement of a medical board certification
- investigating opportunities to increase the number of female physicians in medical leadership positions

This article addresses the following questions:

- What are male and female resident physicians’ preferred future workplaces (hospital, ambulatory care, others)?
- In case the residents strive to work in a hospital, it will be analysed which hierarchical position male and female physicians respectively prefer (specialist without leading responsibilities, senior or chief physician). A special emphasis will be laid on the analysis of the impact of occupational self-efficacy expectation on the willingness to work in leading positions.
- Which obstacles (working hours, childcare) are faced during residency and how do they differ between genders?

The interviewed persons have completed four years of postgraduate medical training when they were surveyed. Therefore, they have had deep insights into the reality of the medical profession and, therefore, their career expectations can be considered consolidated.

## 2. Methods

The KarMed-Study’s database consists of annual postal surveys throughout the entire residency of medical students, who were in their “Practical Year” in 2008/2009. All students in their ‘practical year’ in the medical faculties of Erlangen, Giessen, Hamburg, Heidelberg, Cologne, Leipzig, and Magdeburg were approached. The response rate for the baseline survey (T0) was 48% (n=1012). In the following years the rate was 85% and higher. The survey ended in 2015 after seven years of interviews. 

Preferences regarding the preferred workplace and position were gathered using the following question: “which professional position would you like to reach after finishing your postgraduate medical training?” The answer options were:

- Establish a private practice as general practitioner
- Establish a private practice as specialist physician
- Employment as general practitioner in a private practice or a healthcare centre
- Employment as specialist physician in a private practice or a healthcare centre
- Specialist in a hospital
- Senior physician in a hospital
- Chief physician in a hospital
- University-based research work (without clinical work)
- Work outside medical care (health administration, medical journalism etc.)
- Other goal, namely…
- No concrete ideas

We also asked for the specialty chosen for the medical board certification. The study participants could rank their top three preferences. This article is based on the primary preferences.

In Germany, most private practices are open to statutory and privately health insured patients. In hospitals, apart from chief physicians and their deputies, specialists without directorial responsibilities and specialists-in-training constitute the regular staff of a department. 

Study participants were also asked about their preferred working hours after finishing their residency (“which work hour model do you prefer for your occupation after completing your postgraduate medical training?”). They had to choose between four options: “full-time throughout the entire career”, “part-time throughout the entire career”, “a few years full-time followed by part-time” or vice versa “a few years part-time followed by full-time”. 

Depending on their postgraduate medical training facility, residents allocated themselves to a university hospital, a large hospital (=800 beds), a medium (350-800 beds) or small hospital (<350 beds) or a medical practice/ambulatory healthcare centre.

In this study, parents are defined as persons who live with at least one child in their household permanently or most of the time.

The occupational self-efficacy expectation (hereafter: OSEE) is a psychological assessment instrument that aims to give an insight to one’s competencies of coping with difficulties and problems. The OSEE is an important determinant for positive career development and professional success. It is derived from the general self-efficacy expectation developed by Bandura that is a predictor for success in life management and life satisfaction [14].

A scale developed by Abele et al. was used to assess the residents’ occupational self-efficacy expectation. This scale was validated in connection with the longitudinal study BELA-E (Berufliche Laufbahnentwicklung Erlanger Absolventinnen und Absolventen [eines Universitätsstudiums]; professional career developments of [university] graduates in Erlangen) [15]. It consists of six items where interviewees are supposed to specify their degree of agreement on a 5-level scale that ranges from 1 “strongly disagree” to 5 “strongly agree”. The ratings were then summarised to form a total score. Therefore, the OSEE-scale ranges between values of 5 and 30. 

## 3. Results

### 3.1. Study cohort

Approximately two thirds of all survey participants are women. This is similar to the percentage of women in medical education [2]. After four years of post-graduate training male physicians have a median age of 31 years while female physicians have a median age of 30 years. 80% of the survey participants are in long-term relationships. The share of parents rose from 8% (T0) to 29% (T4). For comparison to the general population: at the time of the survey one fifth of all households in Germany had at least one child [16]. The last two features showed no gender disparity. 

#### 3.2. Differing preferences regarding workplace, position and working hours after residency

As shown in table 1 (Tab. 1), 51% of the respondents state that they want to continue their career in a hospital after post-graduate training, whereas 44% prefer a workplace in ambulatory care (p<0.001). The remaining 5% pursue other goals such as a career in research. The percentage of female physicians who prefer ambulatory care is higher than the percentage of male physicians (46% of female physicians vs. 39% of male physicians), however, this difference is not significant. 

There is a major preference shift in women wanting to work in ambulatory care from wanting to establish private practices towards preferring employment. Male physicians still prefer the establishment of their own private practices, although here we observe a trend towards preferring employment as well. For both genders, parenthood and the wish to work part-time increase the preference for ambulatory care work. 

There are stronger gender differences in the hospitals. The proportion of male physicians who want to work in a hospital after receiving their specialist certificate is 1.4 times higher than the proportion of female physicians. This trend reaches a peak when looking at a position as chief physicians: this ratio is even 4.8 times higher. Only 2% of the female physicians are willing to work as chief physician after achieving their medical board certification. In absolute numbers these are 4 out of 186 female physicians. In contrast, the proportion of female physicians who want to work as specialists without leadership responsibilities is 3 times higher than the proportion of male physicians with the same goal (see table 1 (Tab. 1)). 

There are also differences with regard to the preferred work hours (see table 2 (Tab. 2)). Half the female physicians want to work part-time for a few years or their entire career after post-graduate training, whereas this applied to only 14% of male physicians (p<0.001). Overall, the preference to work full-time during the entire career decreased over the years. This rate is still higher for male physicians (50%) than for females (17%) but the decrease over time was stronger in male than in female physicians. For more details about work time preferences see Ziegler et al. [17].

Female physicians are less likely to work in university hospitals than male physicians (24% vs. 31%; n.s.). Children increase this effect. Furthermore, female physicians are less likely to hold a doctorate title than male physicians (56% vs. 47%; p<0.05). 

#### 3.3. Duration and output of post graduate medical education

Quantitative indicators for the effectiveness of post-graduate medical training are its real duration compared to the minimally required training time according to the Specialty Training Regulations, as well as the dropout rate. We assume that respondents who had gone through four years of residency have a realistic estimation of the end of their postgraduate training.

In the KarMed study, approximately 50% deny the statement “I will get all certificates demanded in the Specialty Training Regulations within the prescribed minimum time of residency”. Female physicians disagree more often than male physicians (51% vs. 40%; p<0.05), just as female physicians who are mothers disagree more than male physicians with children (78% vs. 53%; p<0.001).

The main reasons for an assumed prolongation of residency (multiple answers possible) in female physicians are the interrelated issues of childcare (42%) and part-time work (32%). Curricular and organisational problems (e.g. rotation problems through units) are the main reasons stated by male physicians but are also often found among female physicians: 41% of the male physicians stated that there is “insufficient time for gaining the expertise needed to fulfil the residency requirements”. These results are consistent with the findings of Bestmann et al. [18]. There is no statistical relationship between the reasons for an assumed prolongation of residency and the size of the institution in which the post-graduate training is being performed.

Retrospective surveys among medical specialists confirm our ex ante survey data. For instance, 55% of surgeons stated in a survey run by the German Society of Surgery that they did not manage to finish their residency in the minimum time. In university hospitals, the percentage was even higher with 62% of the respondents reporting the same [19]. Gensch [20] and Rhode et al. [21] presented similar results. 

How many physicians break off their residency cannot be estimated by the prospective KarMed study. It can be assumed that people not continuing their residency also no longer participate in the study either. But, this is only one of many possible reasons for dropping out of the study. Apparently, investigations about residency dropout rates are not of great importance in the Medical Associations. Only the Medical Association of Hessen once provided data for 2005: 14% of male and 35% of female physicians had not managed to become specialists after eleven years, which in most cases is twice the minimum length of residency [8], [22].

#### 3.4. Special problems for mothers in postgraduate education

Especially women with children experience long residencies and show high interruption or dropout rates. A survey among all Bavarian physicians four to five years after having received their medical license in 2004 showed that 15% of female but only 1% of male physicians broke off their postgraduate training in order to care for a child [6]. Similar results are presented by Hancke et al. [23] and Hohner et al. [24].

The KarMed study provides ample information on the particular professional challenges faced by mothers caring for children compared to female physicians without children. As stated above, mothers begin their residency later and they need more time to finish it. 43% of female physicians with children obtained their doctorate, while 49% of those without children did (n.s.). There is a difference in work contracts as well: Female physicians with children hold a part-time contract more frequently (59%) than female physicians without children (7%; p<0,001). For 49% of mothers, the contract does not cover the full time of their residency (see also [25]). In addition, mothers have a higher private workload. All these conditions make the coordination of career and private life against the background of frequently varying working hours and shifts in the working schedule much more challenging. 

#### 3.5. Occupational Self-efficacy Expectation and preferred positions in hospitals

Abele argues that not only those contextual factors presented above have an impact on professional forthcoming but also one’s own assessment of personal capabilities and the “willingness to make an effort”. These aspects are represented in the concept of the occupational self-efficacy expectation [9]. 

Table 3 (Tab. 3) shows that the average of OSEE is lower in female than in male physicians. This difference is small but statistically significant (p<0,001). 

We analysed how the contextual factors (parenthood, doctorate, preference for part-time work, size of training institution) and the ‘internal’ factors (gender, OSEE) have an effect on the physician’s willingness to take on leadership responsibilities in a hospital after completing their postgraduate training (work as a senior or chief physician). Two regression models were evolved for this purpose. Only survey participants who reported working in a hospital as their first career choice (see table 4 (Tab. 4)) are included. The odds ratios in model 1 state that the probability to strive for a leading position is considerably higher for male physicians and physicians (male and female) with a doctorate than for female physicians and those physicians without a doctorate. Controlled for the other variables, the probability of aiming to work in a leading position is doubled when physicians achieved their doctorate (p<0.01) and even quadrupled (p<0.001) when the physician is male. This gender difference was already found in the baseline survey of the medical students during their last undergraduate year (“Practical Year”). At that point in time, the proportion of male medical students who aimed to work as senior physicians was 1.5 times higher than for female medical students. With regard to positions as chief physicians, the share of male students aiming at this goal was 5.4 times higher. During the Practical Year (T0 survey) and after four years of post-graduate training (T4 survey), only 2.2% of the female interviewees wanted to work as a chief physician. Among medical students, the preference for work as a specialist without leading responsibilities was 3.8 times higher for female physicians than for males. 

Compared to model 1, model 2 shows that the variance can be better explained when the OSEE is included (difference pseudo-R²=0.047). There is a strong effect of OSEE on the preference for leading positions: the scale has 25 scale points and an increase of one point increases the probability of aiming at leading positions by about 15% (p<0.01).

Furthermore, table 4 (Tab. 4) shows that parenthood, the preference for part-time work and current work in a university hospital do not influence the willingness to work in leading hospital positions, when controlled for the other variables. 

## 4. Discussion

The findings of the study show that differences between female and male physicians remain during four years of residency. This includes disparities in the preferred work position after certification, field of specialisation, occupational self-efficacy expectation, work-hour preferences, and the expected duration of residency. The attitude towards directory responsibilities of female physicians remained stable after graduating from university (at the latest) and is, therefore, not a result of experiences during residency. It can be assumed that females’ life plans are influenced by anticipated parenthood and its consequences more than it is for their male counterparts. Therefore, it does not matter if these women already have children and experience the balancing act between family life and medical work by themselves or if they see it in their private or occupational surroundings. As long as a female physician intends to become a mother, it is likely that she will adjust her career plans accordingly.

The results regarding the OSEE support this assertion. The OSEE is a powerful factor in explaining gender differences, but there is no significant difference in OSEE between women with at least one child and female physicians without children. The OSEE is associated with the willingness to accept leading responsibilities. When comparing female physicians with a high OSEE score to males with a similar score, no differences with regard to the readiness to take over leadership positions were found. In view of these results, we conclude that specific measures should be implemented or, if already existing, improved to strengthen the occupational self-efficacy expectation of female physicians and, therein, increase their willingness to work in leading positions. Possible measures, which Abele recommends as well, are mentoring programs [26]. Especially in Switzerland evaluations of such programs have offered convincing results [27]. Furthermore, specific PhD programs or other measures of support to achieve the doctorate could be helpful, as well as trainings to develop specific social competencies. One should realise, however, that this would correspond to characteristics (for instance assertiveness, competitiveness, decisiveness, severity, independence) that are linked to a male role model and which male physicians are often criticised for. A doctoral degree is important as it is required for obtaining a “habilitation”, which is required to achieve an academic position in Germany. Also, without “habilitation” it is very difficult to become a chief physician even in non-university hospitals. 

As described above, the restraint in career goals exists already before a female physician has children. We assume that the OSEE develops primarily during undergraduate education, when students start to gather insights into the medical profession and experience the prevalent role models. Therefore, the often proposed measures (increasing the supply of kindergarten places and/or part-time positions for female residents) might not improve the OSEE in the short-term. They might have an effect, but indirectly and on the long term. They can demonstrate to the forthcoming generation of female physicians that it is feasible to coordinate private life and work life within the current working conditions [28], [29].

Moreover, the role of fathers should be reconsidered. As long as the partners or spouses are not willing to take over equal duties in child care, the disparity between female physicians with children and male physicians (with and without children) will remain [30], [31]. Female medical students anticipate this private conflict before getting their medical license and solve it by choosing careers that are less challenging [11], [32], [33]. Male physicians should, therefore, be encouraged to make use of the opportunity of taking longer parental leaves. In addition, work schedule models that allow a stronger focus on one’s private life are very important for men as well. Currently, the traditional family role model with the father providing the income and the mother being responsible for child care is still dominant in Germany. This is demonstrated, for instance, by the fact that medical fathers work more hours than childless male physicians. 

Further research should be conducted on the factors associated with low OSEE, for instance learning or private burdens during undergraduate education and how they evolve during under- and postgraduate education. The KarMed-Study can only provide hints in this regard. 

Our study examines the professional futures of female and male physicians. No evidence can be given about whether these future plans will be fulfilled as predicted or not. Nevertheless, our results support the assumption that it will be increasingly difficult to find suitable staff, especially for chief and senior physician positions [34], if they persists as solely full-time jobs. A hospital career should be manageable for male and female physicians even if they have children or attach more value to their private life for whatever reason.

## Funding

The KarMed-study was funded by the German Federal Ministry of Education and Research and the European Social Fund (Funding Codes 01FP0803 and 01FP0804). The National Association of Statutory Health Insurance Physicians supports the study since 2015. The study was approved by the Ethics Committee of the Medical Association of Hamburg (Approval No. PV3063).

## Competing interests

The authors declare that they have no competing interests. 

## Figures and Tables

**Table 1:**
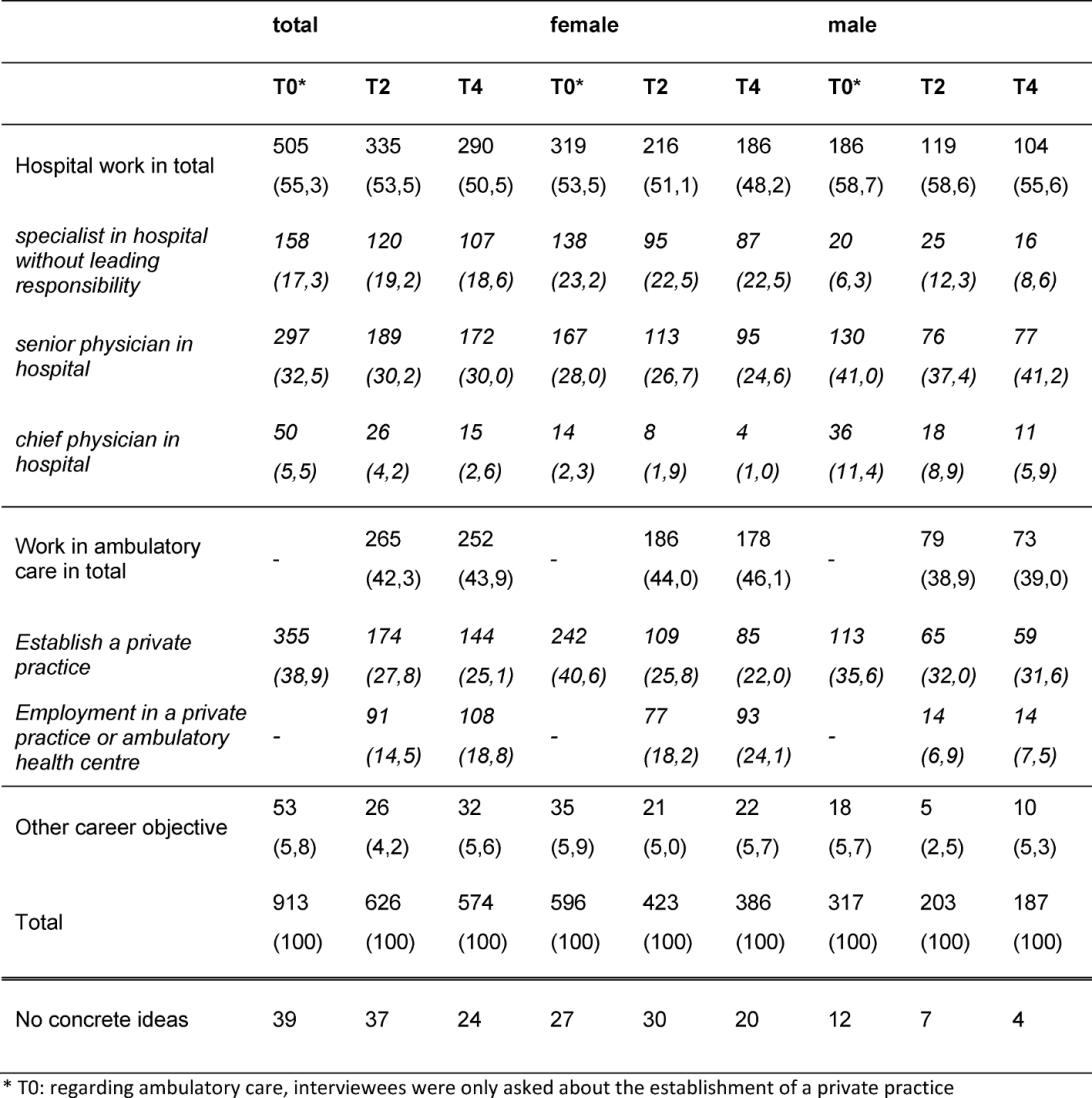
Preferred future workplace (hospital, ambulatory care, others) and position after medical board certification by gender and survey date (absolute frequencies, percentages in brackets)

**Table 2:**
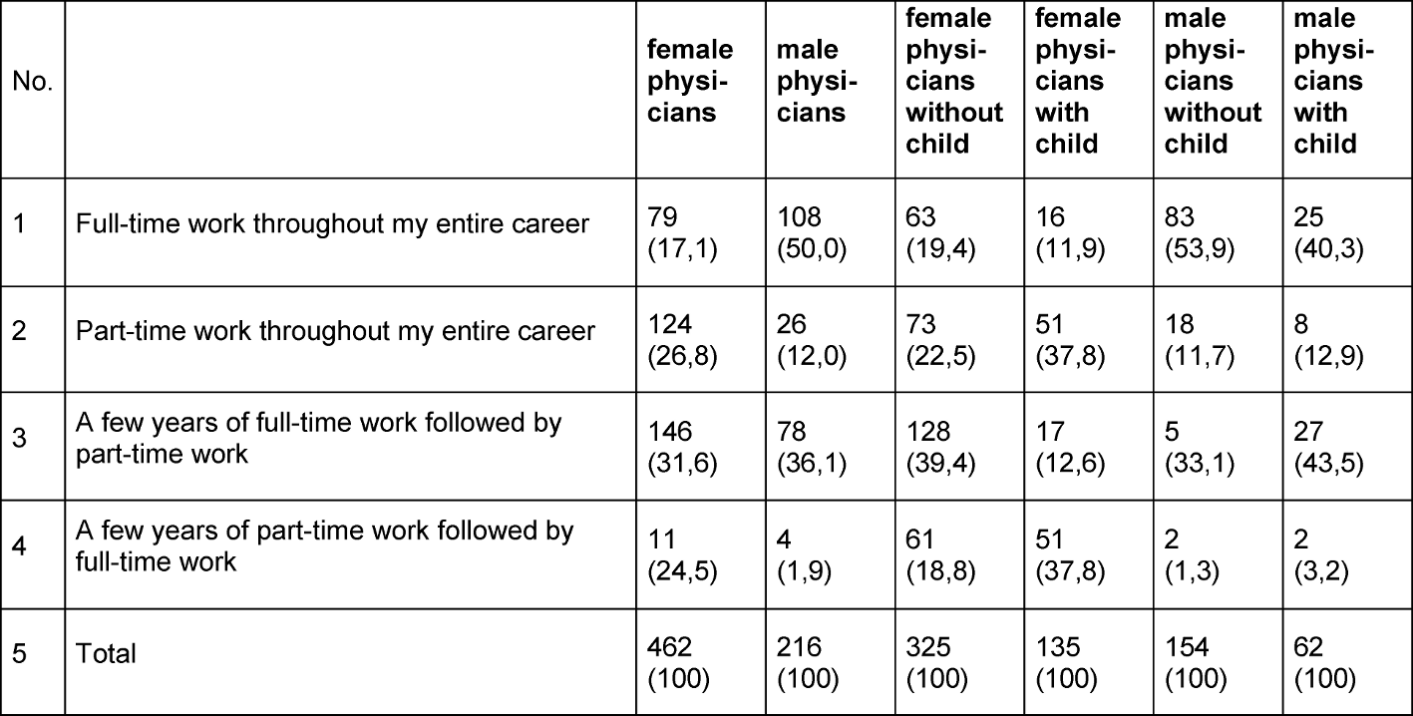
Preferred work time model after medical board certification by gender and parental status (T4; absolute frequencies, percentages in brackets)

**Table 3:**
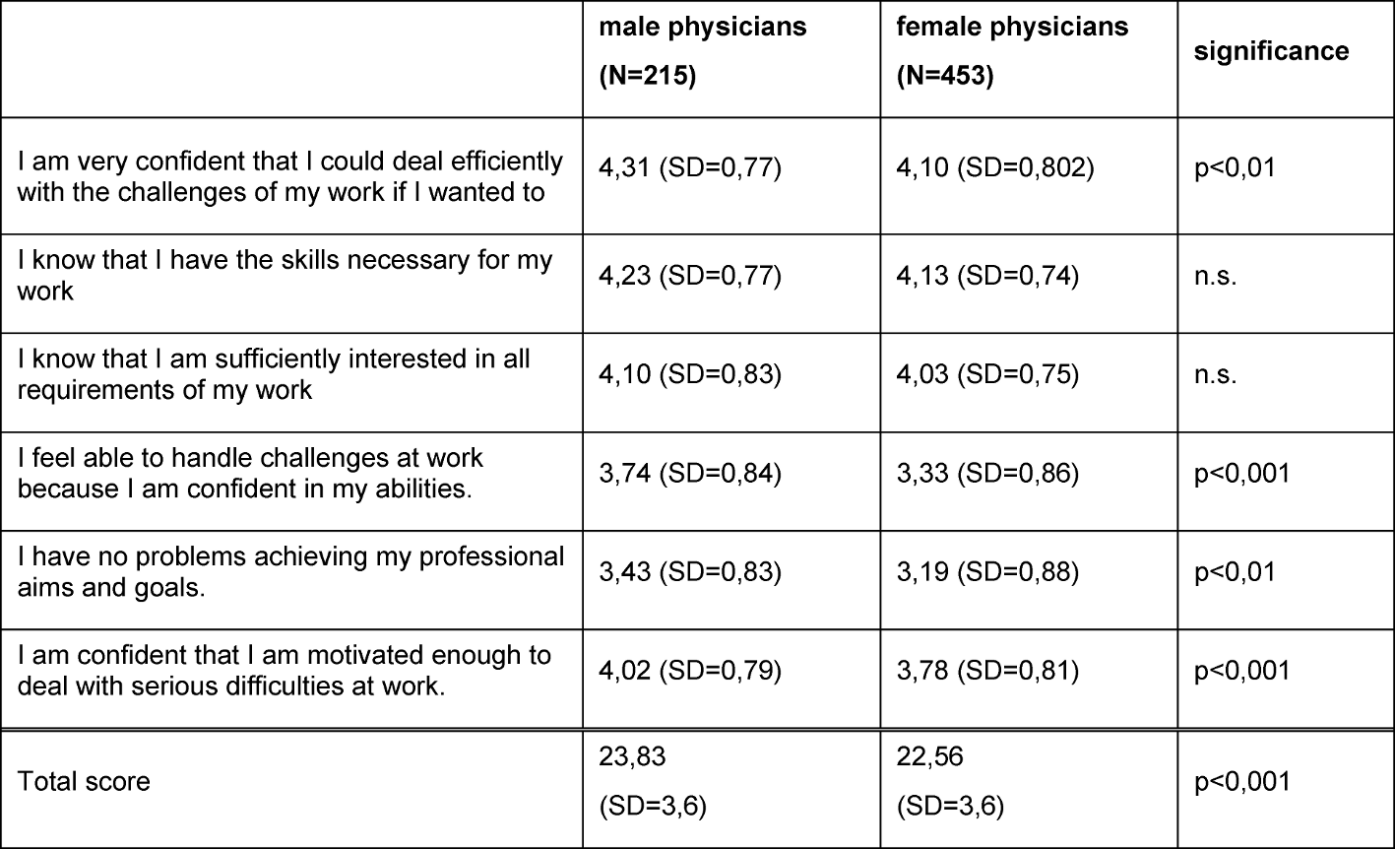
Occupational self-efficacy expectations of male and female physicians after four years of residency (item averages and standard deviation [SD])

**Table 4:**
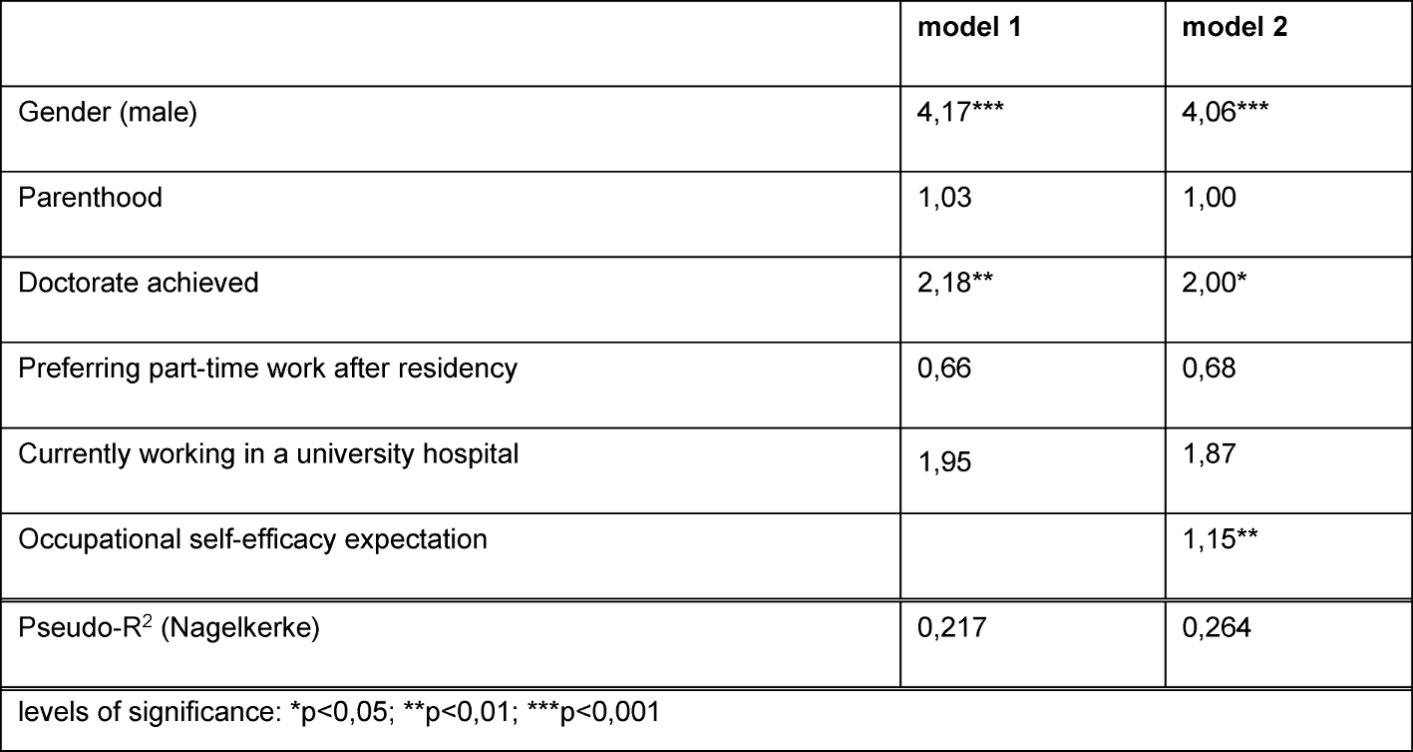
Logistic regression: Odds rations regarding the preference to work in a leading position in a hospital as the dependent variable (N=290, physicians who prefer to work in a hospital after residency)
